# When your pain signifies my gain: neural activity while evaluating outcomes based on another person’s pain

**DOI:** 10.1038/srep26426

**Published:** 2016-05-19

**Authors:** Fang Cui, Xiangru Zhu, Ruolei Gu, Yue-jia Luo

**Affiliations:** 1 Institute of Affective and Social Neuroscience, Shenzhen University, Shenzhen, China; 2 Institute of Cognition and Behavior, Henan University, Kaifeng, China; 3 Institute of psychology, Chinese Academy of Sciences, Beijing, China; 4 Research Center of Sport Psychology, Wuhan Sports University, Wuhan, China

## Abstract

The overlap between pain and reward processing pathways leds researchers to hypothesize that there are interactions between them in the human brain. Two hypotheses have been proposed. The “competition hypothesis” posits that reward can reduce pain-related neural activity and *vice versa*. The “salience hypothesis” suggests that the motivational salience of pain and reward can be mutually reinforced. However, no study has tested these two hypotheses from temporal perspective as we know. In the present study, pictures depicted other people in painful or non-painful situations were used to indicate the valence of outcomes in a gambling task. The event-related potential results revealed an interaction between another person’s pain and outcome valence in multiple time stages. Specifically, the amplitudes of the N1 and P3 were enhanced in the win condition compared with the loss condition when the outcome was indicated by painful picture. This interactions between pain and reward support the salience hypothesis but not the competition hypothesis. The present results provide evidence from human subjects that support the salience hypothesis, which claims that observing other people’s pain can enhance the salience of reward.

Pain and reward are both powerful motivators for human behavior. Noxious stimuli that trigger painful experiences induce avoidance behavior, whereas stimuli that predict reward induce approach behavior^2^. These behavioral tendencies have historically been considered opposite to each other^3^. Partly due to this reason, the neural mechanism of reward processing and that of pain processing have been investigated largely independently^4^. However, emerging brain-imaging evidence suggests that overlapping brain regions are recruited in the processing of both painful and rewarding stimuli, including the lateral prefrontal cortex, anterior insula (AI), posterior insula, orbitofrontal cortex, medial prefrontal cortex, anterior cingulate cortex (ACC), ventral striatum, and amygdala, among others^3^. The extensive similarities in the neural substrates of pain and reward systems encourages investigators to discuss the interactions between the two^5^.

Two competing theories have been proposed to describe interactions between the reward and pain systems. The “competition hypothesis” proposes that reward can reduce pain-related neural activity and *vice versa*^6^. According to this theory, reward and threat (mostly referring to physical pain) compete against each other. In situations that involve both reward and threat, responses to reward are reduced by threat-related processing and *vice versa*. In previous functional magnetic resonance imaging (fMRI) studies, participants were exposed to pictures of their significant others (i.e., highly rewarding stimuli) while experiencing physical pain (i.e., highly salient but negative stimuli). A reduction of neural activity was detected in regions that are related to the affective component of pain (ACC, AI) compared with participants who were exposed to pictures of neutral objects while experiencing physical pain^7^,^8^. In another study, participants underwent a task in which they were simultaneously told about the chance of winning money (reward) and the chance of receiving a shock (pain). The study found that the effect of reward was reduced by threat and *vice versa*^9^. The “salience hypothesis” suggested that rewarding and painful stimuli are represented in terms of their motivational salience. In situations that involve both reward and threat (pain), neural activation would be enhanced compared to the “single” condition because of aggregated motivational salience^9^. Interestingly, although the salience hypothesis has been extensively discussed, little empirical evidence has been provided by human studies to support this theory^9^.

In experimental paradigms that were used in most previous studies that devoted to investigate the interaction between reward and pain, rewarding stimuli were task-relevant and painful stimuli were used as distractors, or the reverse was true. This might lead to competitions on limited attentional and cognitive resources, which may provide experimental results in favor of the competition hypothesis^6^. Additionally, most studies in this field used a cue paradigm, in which a cue that indicates subsequent pain or reward experiences was presented to the participant. After a time delay, the actual painful or rewarding stimulus was presented. This paradigm allowed the participants to weigh between pain and reward during the time delay, which may trigger a competition between the subsequent processing of pain and that of reward^9^. However, what remains largely unknown is whether the processing of these two attributes would be weakened when the same stimulus contains both painful and rewarding information (according to the competition hypothesis) or whether the salience of this stimulus is enhanced as a whole (according to the salience hypothesis). Clarifying this issue is crucial because it would exclude the possibility that participants make a “trade-off” between pain and reward. In the present study, participants performed a gambling task, in which pictures that showed other people in painful or non-painful situations were used to indicate the valence of their monetary outcome (i.e., win or loss). Specifically, in half of the trials, a picture that showed another person’s pain indicated monetary gain in the current trial. In the other half of the trials, a picture that showed another person’s pain indicated monetary loss. In this task design, rewarding information could be directly conveyed by painful information, thus excluding the possibility of competition on attentional resources between reward processing and pain processing.

It is worth noting that the salience hypothesis and competition hypothesis are not antagonistic to each other. One possibility is that they are both true, but manifest in different temporal stages of processing. To our knowledge, the literature lacks high-temporal-resolution data that could directly address this possibility. In the present study, the event-related potentials (ERPs) were recorded to investigate the interaction between pain and reward from a temporal perspective. Using ERPs with millisecond temporal resolution is beneficial for detecting dynamic changes in the interaction between pain and reward, thus may provide novel knowledge about the debate^10^.

Painful stimuli are herein referred to as pictures that show another person’s pain. Seeing another person suffering pain has been consistently reported to be an aversive and unpleasant experience^11^,^12^. Results from fMRI studies regarding the perception of another person’s pain found that when observing such pain, brain regions that are involved in self-pain experience (e.g., the AI and ACC) were activated, which means when observing other’s pain, we partly experienced pain oursevleve^13^,^14^,^15^. This ability to feel and understand how other peoples feel when observe other’s experience the same feelings (e.g, pain) was named “empathy”^16^,^17^. Neuroimaging evidence suggests that two components (i.e., affective and cognitive) are involved in perceiving another person’s pain, which are subserved by distinct brain networks^18^. The affective component of empathy has been framed as reflecting rapid, bottom-up activation of subcortical/cortical circuitries^19^,^20^,^21^,^22^. The cognitive component of empathy has been shown to be influenced by higher-level, top-down signals that originate in prefrontal cortical circuitries^18^,^21^. Event-related potential studies support this two-system model, in which both early (N1, N2) and late (P3) ERP components are involved when a subject observes another person receiving painful stimuli compared with non-painful stimuli. These findings indicate that the perception of another person’s pain generally involves three key processes: an early, automatic, bottom-up process that is related to perception-action coupling (indexed by the N1 component^23^); an early affective arousal and sharing process (reflected by the N2 component); and a later, cognitively controlled, top-down process (indexed by the P3 component^24^,^25^,^26^.

Rewarding stimuli are herein referred to as monetary outcomes that are indicated by pictures. The fast and accurate representation of outcome feedback is crucial for reward processing^27^.Human brain has been shaped by evolutionary adaptation into developing specific mechanisms to assess the valence, magnitude, and other aspects of outcomes, thus linking outcome information with subjective and motivational significance^28^. Event-related potential studies have found that a centro-parietal located P3 component encodes the salience of stimuli^29^,^30^,^31^. Positive outcomes evoke a larger P3 than negative outcomes, and outcomes with a larger magnitude elicit a larger P3 than outcomes with a smaller magnitude^32^,^33^.

We compared ERPs when the participants observed pictures that showed another person’s pain or no pain, which indicated different kinds of monetary consequences of gambling. Our hypothesis was that when a positive outcome (reward) was represented as another person’s pain, the competition hypothesis would be supported if the reward value reduces neural response to painful stimuli. Conversely, the salience hypothesis would be supported if the reward value enhances neural response to painful stimuli. More specifically, if the “Pain-Win” (painful picture indicates monetary win) condition elicits larger amplitudes of ERP components compared with other conditions, particularly the “No Pain-Win” condition, then the interaction between pain and reward during this stage fits the salience hypothesis. Otherwise the interaction during this stage fits the competition hypothesis. Furthermore, if the experimental effect described above occurs in the early visual discrimination and/or attentional process, then the effect should be observed on the N1 component; if this effect mainly modulate the affective arousal of painful stimuli, then we would observe the effect on the N2 component; finally, if the effect influences the later cognitive evaluation process, we would expect to find the effect on the P3 component as well.

## Materials and Methods

### Participants

Twenty-three right-handed university students with no history of neurological disorders, brain injury or developmental disabilities were recruited through advertisement to participate in the experiment. The handedness of participants was tested using the Chinese translation of Edinburgh Handedness Inventory^34^. All of them have normal or corrected to normal vision. The histories of neurological disorders of the participants were assessed through a self-report version of questionnaires. In the questionnaire, the participants need to answer YES or NO to report if they have ever been diagnosed as following conditions: stroke, seizure, anxiety, depression, other neurologic disorder (nerve, spinal cord or brain disorder), significant vision or hearing disorders. We only recruited participants who gave no YES to any condition. All participants signed an informed consent form before the experiment. The experiment was conducted in accordance with the Declaration of Helsinki and was approved by the Medical Ethical Committee of the Medical School of Shenzhen University, China. The data of three subjects were excluded; two of them were excluded because too few epochs survived after artifact correction (less than 50%) and one was due to extremely biased behaviors in the game (consistently choosing the large value option in all trials). As a result, the final sample consisted of twenty participants (11 male, 22.95 ± 0.44 years (mean ± S.E)).

### Stimuli

**Figure 1:**
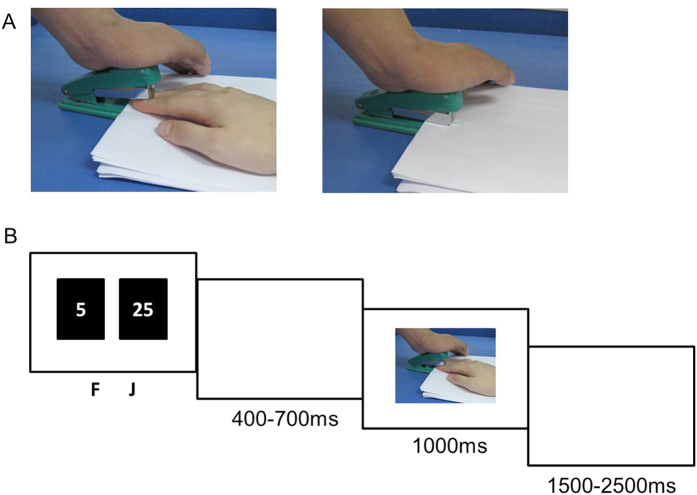
Experimental design. (**A**) an example of the pictures used in the experiment. The Left side shows a painful picture and the right side shows a non-painful picture. (**B**) An example of a single trial.

The stimuli used in the experiment were pictures showing a person’s hands/forearms/feet in painful or non-painful situations, which have been used in previous ERP studies^1^,^35^. All the situations depicted in these pictures were ordinary events in daily life. All the events showing in the non-painful pictures were corresponding to those in the painful pictures, but without the nociceptive component (Fig. 1A). There were 60 painful pictures and 60 non-painful pictures in total. All of them had the same size of 9 × 6.76 cm (width × height) and 100 pixels per inch. Luminance, contrast ratio and color were matched between painful and non-painful pictures. Previous studies have confirmed that painful and non-painful pictures were significantly different on the dimensions of pain intensity and arousal level, but not emotional valence, according to self-reported rating^35^.

### Experimental procedures

Stimulus display and behavioral data acquisition were conducted using E-Prime software (Version 2.0, Psychology Software Tools, Inc, Boston, USA). During the task, participants sat comfortably in an electrically-shielded room approximately 100 cm from a 15 inch LCD color computer screen. Each trial began with the presentation of two grey rectangles (2.3° × 3.2° of visual angle) on the left and right sides of a fixation point in which two numbers (“25” and “5”, indicating the gambling points) were individually presented to indicate two alternative options. The positions of the two numbers were counterbalanced across trials. The participants were asked to make a selection by pressing the “F” or “J” key on a keyboard with their index fingers. The rectangles remained on the screen until a response was given. Then a blank interval would present for 400 to 700 ms randomly. After that, the outcome of the participants’ choice was presented as pictures showing a person’s hands/forearms/feet in painful or non-painful situations. The picture would last for 1000 ms on the screen. In two of all four blocks a picture showing others in painful situations indicated monetary loss in the current trial, while pictures showing others in non-painful situations indicated monetary gain (other’s pain = self’s loss). For example, if participants chose “5” and received a painful picture, that means they had loss 5 points in this trial. In the other two blocks pictures showing others in pain indicated monetary gain while pictures showing others in non-painful situations indicated monetary loss (other’s pain = self’s gain). In this condition, a painful picture after selecting “5” means the participants had loss 5 points in this trial. There was an interval of 1500 to 2500 ms between trials (Fig. 1B). There were 120 trials in each block. The order of the blocks was counterbalanced using an ABBA.

The experiment was a 2 × 2 × 2 within-subjects design. The first factor was the content of pictures: painful or non-painful. The second factor was the magnitude of the choices: small (5) or large (25). The third factor was the valence of the outcome: win or lose. Thus, there were 8 conditions in total (Table 1).

Before the experiment, participants were instructed about the rules and the meaning of the pictures in the task. In addition, they were told that the higher the score they earned, the more bonus money they would receive at the end of the experiment. However, after the participants finished the task, they were briefed that their total gains and losses were predetermined, and all participants received 150 RMB (~25 US dollars) for their participation.

### EEG acquisition and analysis

Electroencephalography (EEG) data were recorded from a 63-electrode scalp cap using the 10–20 system (Brain Products, Munich, Germany). The channel at the middle site between FPz and Fz (i.e., the location of FCz) was used as reference. Two electrodes were used to measure the electrooculogram (EOG). EEG and EOG activity was amplified at 0.01 Hz~100 Hz band-passes and sampled at 500 Hz. All electrode impedances were maintained below 5 kΩ.

EEG data were pre-processed and analyzed using Matlab R2011b (MathWorks, Natick, USA) and EEGLAB toolbox (EEGLAB v13.4.3b)^36^. EEG data at each electrode were down-sampled to 250 Hz. Then the EEG signals were-referenced to the grand average and the electrode FCz was reinstated. After that the signals were filtered with 0.01–30 Hz band-pass. The time windows from 200 ms before to 1000 ms after the onset of picture were segmented. All epochs were baseline-corrected with respect to the mean voltage over the 200 ms preceding the onset of stimulus. EOG artifacts were corrected using an independent component analysis (ICA)^37^. Epochs with amplitude values exceeding ±50 μV at any electrode were excluded from the averaging.

Further statistical analysis was conducted in IBM SPSS Statistics 22 (IBM Corp., Armonk, USA). Statistical analysis was conducted at electrodes selected from the frontal (Fz, FCz, F3-F4, FC3-FC4), central (Cz, CPz, C3-C4, CP3-CP4), parietal (Pz, P3-P4), temporal (T7-T8, TP7-TP8, P7-P8) and occipito-temporal (POz, Oz, PO3-PO4, PO7-PO8)^24^. The main concern of the current research is the interaction between the “Picture” factor and the “Outcome” factor. We did not include the factor “Value” (small/large) in the Repeated measures ANOVA. Instead, the repeated measures ANOVA (2 (Picture: Painful/Non-Painful) × 2 (Outcome: Win/Lose) × 5 (Regions: frontal/central/ parietal/temporal/occipito-temporal) was performed for each component. Mean amplitudes were obtained from the grand-averaged waveforms. The time windows for ERP amplitude analysis were set as below: from 90 to 140 ms for the N1 component;; from 200 to 240 ms for the N2 component and from 480 to 680 ms for the P3 component. Degrees of freedom for F-ratios were corrected according to the Greenhouse–Geisser method. Statistical differences were considered significant at *p* < 0.05; post-hoc comparisons were Bonferroni-corrected at *p* < 0.05. Only significant effects were reported for the sake of brevity.

## Results

### Behavioral data

The percentage of trials in which participants selected the large option (25) and small option (5) were 53 ± 6.1% and 46 ± 7.1%, respectively. The average reaction time was 890.03 ± 56.45 ms. The mean RTs and choices in different condition are shown in Table 2. ANOVAs of RTs and number of trials show no significance between conditions.

### ERPs

The grand averaged ERPs elicited by pictures were computed separately for each condition. The grand averaged ERPs for the pictures displayed a negative component from 90 to 140 ms (N1) over the frontal and central regions, a negative deflection from 200 to 240 ms (N2) over the central and parietal region and a late positive component from 480 to 680 ms (P3) over the central and parietal regions.

**Figure 2:**
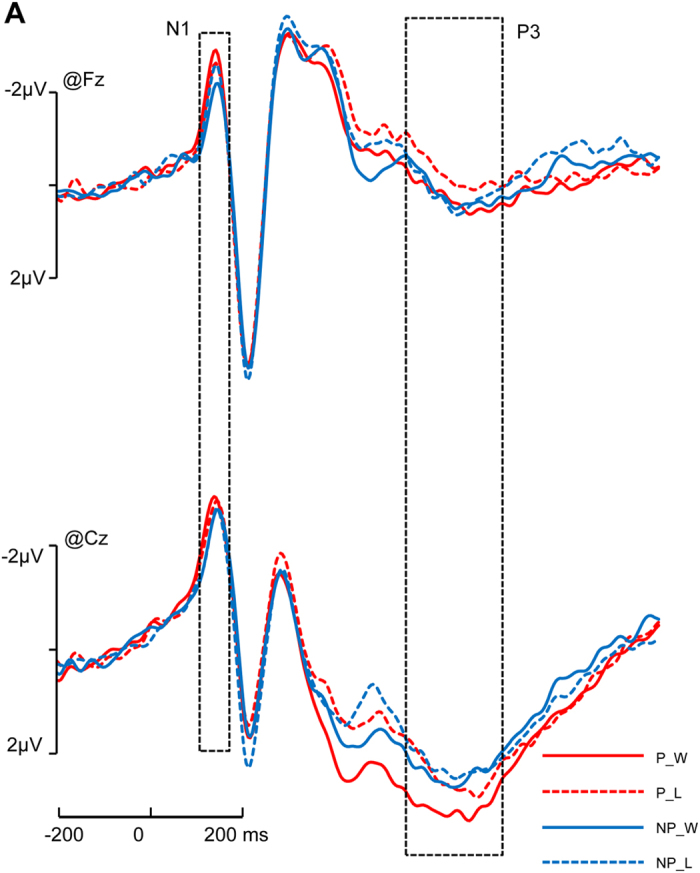
Interaction on N1 and P3. ERPs under 4 conditions (P_W: Painful picture and Win; P_L: Painful picture and Lose; NP_W: Non-painful picture and Win; NP_L: Non-painful picture and Lose).

For the N1 component, we observed a significant main effect of the region (*F* (4, 76) = 35.618, *p* < 0.001, η_p_^2^ = 0.625). The N1 component was larger in the frontal and central regions than in the parietal, temporal, and occipito-temporal regions. We also observed a significant interaction of picture × outcome interaction (*F* (1, 19) = 7.173, *p* = 0.015, η_p_^2^ = 0.274). By using pairwise comparisons, we found that only when the picture was a painful one, “win” elicited a more negative peak than “loss” (*p* = 0.048); however, when the picture was a non-painful one, the difference between win and loss was insignificant (*p* = 0.156) (Fig. 2). The mean amplitudes in different condition are shown in Table 3.

For the N2 component, we observed a significant main effect of the region (*F*(4, 76) = 14.100, *p* < 0.001, η_p_^2^ = 0.426). The N2 component was most prominent in the central and parietal regions. We also observed a significant main effect of the picture (*F*(1, 19) = 9.061, *p* = 0.007, η_p_^2^ = 0.323); painful pictures elicited a more negative peak than non-painful pictures on this component. A significant interaction of picture × region was also detected (*F*(4, 76) = 9.026, *p* < 0.001, η_p_^2^ = 0.322). Pairwise comparisons showed that the difference between painful and non-painful pictures on this component were significant in the central, parietal, and temporal regions (*p* = 0.02, *p* < 0.001, *p* = 0.08, respectively).

**Figure 3:**
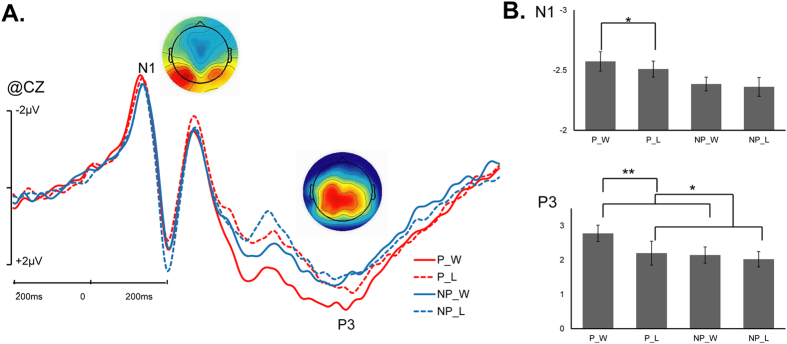
Cortical responses to picture stimuli. (**A**) ERPs elicited by the painful and non-painful pictures indicating win and lose at CZ site. The voltage topographies illustrate the scalp distribution of each component; (**B**) the averaged amplitudes within the N1 and P3 time Window in each conditions (P_W: Painful picture and Win; P_L: Painful picture and Lose; NP_W: Non-painful picture and Win; NP_L: Non-painful picture and Lose) (**p < 0.01; *p < 0.05).

On the P3, we observed a significant main effect of the region (*F*(4, 76) = 16.642, *p* < 0.001, η_p_^2^ = 0.467); the P3 component was demonstrated in the central and partial areas. We observed a significant main effect of the picture (*F*(1, 19) = 43.792, *p* < 0.001, η_p_^2^ = 0.697). We also found a significant three-way interaction of picture × outcome × region (*F*(4, 76) = 3.828, *p* = 0.026, η_p_^2^ = 0.168) (Fig. 3A,B). Pairwise comparisons showed that only when the picture was painful the difference between win and loss were significant in the frontal, central, temporal, and occipito-temporal regions (Fig. 2); in these regions, only when the picture was a painful one, win elicited significant larger amplitudes of the P3 than loss (*p* = 0.002 for frontal, *p* = 0.002 for central, *p* = 0.006 for temporal, *p* = 0.030 for occipito-temporal, respectively (Fig. 3C,D)).

There was no other significant main effect or interaction (*p* > 0.066). The mean amplitudes in different condition are shown in Table 3.

## Discussion

To temporally explore interactions between viewing another person’s pain and monetary outcome evaluation, the present study utilized the ERPs. Our design allowed us to measure ERP responses to both painful and rewarding stimuli, thus is suitable to probe stimulus-dependent processes that are immune to the trade-off between each other. Analysis of the ERPs during the presentation of painful and non-painful pictures in win and loss conditions revealed a significant interaction between another person’s pain and the outcome valence, which was reflected by a larger N1 component in the win condition than in the loss condition when the reward was indicated by painful pictures. For the P3 component, the interaction between the two factors was also significant, reflected by larger amplitude in the win condition than in the loss condition when the outcome was indicated by a painful picture. Overall, our findings support the salience hypothesis but not the competition hypothesis. In brief, when reward and pain were jointly presented, reward processing was enhanced by painful information.

The N1 is an early sensory component that is involved in selective attention and motivational processing^38^, which are both sensitive to the processing of reward and another person’s pain. For example, positive emotional words evoke larger N1 amplitudes than negative words. Stimuli with high motivational salience have also been reported to evoke larger N1 amplitudes, such as game rewards for pathological computer game players compared with healthy players^39^ and immediate rewards for hypomania patients compared with controls^40^. Additionally, the processing of another person’s pain can also modulate the N1 component, indicating that pain processing occurs during this stage^24^,^41^,^42^. The engagement of the N1 in the processing of both reward and another person’s pain provides a basis for the interaction between them. For the N1 component, we observed a significant interaction between the picture and outcome. Specifically, only when the picture was painful, the win condition elicited a more negative peak than the loss condition. This data pattern indicates that participants were more sensitive to outcome valence when a painful picture was used as the outcome indicator, which clearly supports the salience hypothesis but not the competition hypothesis. This result is also consistent with previous findings in animal studies, that is, when animals confronted a reward in a conflict situation (i.e., a reward followed by an electric shock), they tended to be more sensitive to the reward^43^. In our opinion, the theoretical significance of the interaction on the N1 can be twofold. This phenomenon indicates that the interaction between pain and reward occurs at an early processing stage, which provides valuable temporal information to the field of studies.

The N2 component has been consistently reported in observing other’s physical pain and it was suggested to index an early automatic component related to the sensitivity to other’s pain^44^, as well as a biomarker of the affective component of empathy for pain^44^. In the current experiment, we have found a main effect of picture on the N2, such that painful pictures elicited significant larger amplitudes of the N2 than non-painful pictures. This result is consistent with the literature that painful stimuli trigger higher level of affective arousal in the observers than the non-painful stimuli^45^.

As mentioned in the Introduction above, the P3 component is related to the processing of both reward and another person’s pain. Generally, “win” conditions evoke a larger P3 than “loss” conditions (for a review, see^46^). Viewing a picture of another person in pain also evokes a larger P3 than viewing a picture of another person who is not in pain^1^,^24^,^35^,^35^,^47^. The engagement of the P3 in both kinds of information processing provides a basis for the interaction between them. For the P3 component, the main effects of picture and outcome were both significant. In addition, we found a significant picture × outcome interaction similar with the N1, in which the “win” condition evoked a larger P3 than the “lose” condition only when the picture was painful. Together with the N1 results, this result supports the salience hypothesis but not the competition hypothesis during the later cognitive processing stage. That is, the processing of monetary win was enhanced by the processing of pain. The P3 is widely regarded as a measure of motivational significance^32^,^48^,^49^,^50^. Therefore, the P3 pattern supports the main point of the salience hypothesis that neural activations in conditions that involve both reward and pain significantly enhance because of aggregated motivational salience.

Notably, both the early N1 and late P3 showed significant interactions between pain and reward. For both the N1 and P3, when positive outcome was indicated by a painful picture, their amplitudes were enhanced, which supports the salience hypothesis. However, the present results appear to be inconsistent with previous fMRI studies that generally supported the competition hypothesis. Differences between the present experimental design and that in previous studies may explain this inconsistency. In task designs of many previous studies, painful information and rewarding information were allocated to separate stimuli, only one of which was set to be task-relevant. Thus to finish the task, participants have to focus on one attribute while suppress the processing of the other. It is not surprising that this kind of task design leads to experimental results in favor of the competition hypothesis. In contrast, the rewarding information and painful information in the present study were combined into one stimulus, and both attributes are set to be task-relevant. The present results reveal that when there is no competition for cognitive resources, stimuli that have both painful and rewarding attributes have the highest level of motivational salience, which support the salience hypothesis rather than the competition hypothesis.

### Limitations

In our opinion, there were two major limitations of the present study. First, in the present study, we used painful pictures to present the “pain” concept. Although numerous studies support that observing other people in pain could elicited both somatosensory and affective components of the subjective pain experience^19^,^51^,^52^,^53^, further research is still necessary to examine the reliability of current findings with first-hand painful stimuli instead of the “observed pain”.

Second, painful pictures were paired with either win (i.e., reward) or loss (punishment) outcomes in the present study, but we did not contain a condition in which joyful pictures were paired with win or loss outcomes. However, to provide a full and appropriate test of competing hypotheses of interactions between reward and pain processing, the usage of positive emotional rather than neutral images paired with win or loss outcomes is needed. Instead, the present study only partially evaluates the competing hypotheses.

## Additional Information

**How to cite this article**: Cui, F. *et al.* When your pain signifies my gain: neural activity while evaluating outcomes based on another person’s pain. *Sci. Rep.* **6**, 26426; doi: 10.1038/srep26426 (2016).

## Figures and Tables

**Table 1:** Conditions.

Conditions	Picture	Outcome	Condition name
1	Painful	Win	P_W
2	Non-Painful	Lose	NP_L
3	Painful	Lose	P_L
4	Non-Painful	Win	NP_W

There were three factors (the chosen Value, Picture and Outcome), each including 2 levels resulting 8 conditions in total. However, since the factor “Value” was not included in later analysis, only two other factors were considered resulting 4 conditions as listed.

**Table 2:** Behavioral data.

	Small bet (5)		Larger bet (25)	
	Pain = Win	Pain = Lose	Pain = Win	Pain = Lose
RTs (ms)	876.93 ± 45.22	902.61 ± 65.02	899.23 ± 39.12	881.33 ± 67.53
Number of trials	110.2 ± 14.22	112.54 ± 21.22	124.95 ± 25.22	124.31 ± 15.10

Mean RTs (ms) and choice. There were four conditions here: choosing small bet when painful picture representing win; choosing small bet when painful picture representing lose; choosing large bet when painful picture representing win and choosing large bet when painful picture representing win.

**Table 3:** ERP data.

Condition	Region	Amplitudes of N1 (mean ± SD); (μV)	Amplitudes of P3 (mean ± SD), (μV)
P_W	Frontal	−1.93 ± 1.14	1.02 ± 1.59
	Central	−1.17 ± 0.97	2.72 ± 1.13
	parietal	0.99 ± 1.24	1.63 ± 2.16
	temporal	1.30 ± 1.03	−1.34 ± 0.90
	occipito-temporal	2.77 ± 1.64	−0.67 ± 2.24
P_L	Frontal	−1.79 ± 1.22	0.61 ± 1.59
	Central	−1.25 ± 0.87	2.35 ± 1.09
	parietal	0.67 ± 1.76	1.71 ± 1.94
	temporal	1.38 ± 1.00	−1.02 ± 1.08
	occipito-temporal	2.51 ± 1.61	−0.29 ± 1.68
NP_W	Frontal	−1.61 ± 1.08	0.92 ± 1.46
	Central	−1.21 ± 0.88	2.32 ± 1.02
	parietal	0.70 ± 1.80	1.06 ± 1.84
	temporal	1.17 ± 0.96	−1.12 ± 0.96
	occipito-temporal	2.52 ± 1.67	−0.74 ± 1.13
NP_L	Frontal	−1.80 ± 1.16	0.95 ± 1.08
	Central	−1.08 ± 1.02	2.19 ± 1.06
	parietal	1.05 ± 1.72	1.97 ± 0.85
	temporal	1.20 ± 1.27	−1.01 ± 0.94
	occipito-temporal	2.76 ± 1.61	−0.86 ± 1,09

The means and SDs of N1 and P3 amplitudes in all conditions.
